# Evaluating the agreement of ultrasound imaging and beta-human chorionic gonadotropin (β-hCG) measurement in confirming completed medical abortion: cross-sectional study

**DOI:** 10.5339/qmj.2021.22

**Published:** 2021-07-12

**Authors:** Mahboubeh Peracheh, Batool Teymouri, Narjes Noori, Taraneh Arbabzadeh, Marzieh Ghasemi

**Affiliations:** ^1^Department of Obstetrics and Gynecology, Zahedan University of Medical Sciences, Zahedan, Iran; ^2^Department of Obstetrics and Gynecology, Pregnancy Health Research Center, Zahedan University of Medical Sciences, Zahedan, Iran E-mail: drghasemi@zaums.ac.ir; drghasemim@yahoo.com; ^3^Department of Obstetrics and Gynecology and Perinatology, Shohaday Tajrish Hospital, Shahid Beheshti University of Medical Sciences, Tehran, Iran

**Keywords:** Serum β-hCG, Transvaginal ultrasound, Complete medical abortion

## Abstract

Objective: Clinical methods that are generally used to evaluate the completeness of medical abortion are not reliable. Ultrasound imaging and beta-human chorionic gonadotropin (β-hCG) measurements are used to diagnose completed medical abortion, but a precise evaluation of these two methods has shown contradictory results. The purpose of this study is to evaluate the agreement of serum β-hCG measurement and ultrasound imaging to confirm complete medical abortion.

Materials and Methods: This study was conducted on pregnant women who had been referred to our center for medical abortion from 2015 to 2017. All cases occurred in the first trimester of pregnancy. They obtained one or two doses of vaginal misoprostol for medical abortion. Success rate of medical abortion was measured by both transvaginal ultrasound imaging and consecutive serum β-hCG measurements two to four weeks after initial treatment.

Results: Among the 275 women who completed the study, complete medical abortion was confirmed by serum β-hCG in 231 women (84.3%) and transvaginal ultrasound imaging in 195 women (70.8%) after two weeks. All remaining cases completed the medical abortion after an additional two weeks, confirmed by both transvaginal ultrasound imaging and serum β-hCG. The sensitivity, specificity, positive, and negative predictive values of β-hCG were 95.2%, 86.7%, 84%, and 70%, respectively; and these values for transvaginal ultrasound imaging were 68.5% 64.5%,77%, and 30.%, respectively, for the diagnosis of completed medical abortion.

Conclusion: Serum β-hCG measurement is as effective as transvaginal ultrasound imaging to confirm successful medical abortion in early pregnancy.

## Introduction

Abortion is the spontaneous death of an embryo or fetus before survival and is also a common early pregnancy complication that could have effects, such as depression and anxiety.^1,2^ There are approximately 56 million abortions in the world every year.^3^

In Iran, the rate of abortion is approximately 22%.^4^ Factors that increase the risk of abortion include increased maternal age, prior abortion, exposure to cigarette smoke, obesity, diabetes, thyroid disorders, and drug or alcohol use.^1^ Abortion procedures are categorized into two main types of traditional and medical/surgical methods.^3,5^ In traditional methods, healers use medications to perform abortions; for instance, a research in Guatemala has shown that 49% of abortions are performed by traditional experts.^6^ Similarly, a recent study from Tanzania found that almost half of the women who have been hospitalized after an unwanted abortion have resorted to a provider who uses traditional methods to induce miscarriage.^7^ Anthropologists who have studied the prevention of pregnancy and abortion in non-Western societies have reported the use of a large number of plant specimens but have rarely paid attention to their side effects.^8^

Surgical or medical-based procedures may be the latest methods of abortion. Surgical procedures are based on cervix dilation and cervical discharge by curettage. Medical abortion procedures, sometimes referred to as “non-surgical abortion,” are ways in which medications are used for abortion.^3^ This type of abortion sometimes involves the use of two drugs: mifepristone and misoprostol. The doctor or nurse will recommend a schedule, and the woman should take the second prescription, misoprostol, less than 48 hours after taking mifepristone. Mifepristone prevents pregnancy development and misoprostol empties the uterus, which starts one to four hours after taking the drug. As the uterus empties, the patient will experience congestion and bleeding, which may feel like an unusual, heavy menstruation. Some women feel tighter than others. Approximately four to five hours, the pregnancy tissue may have passed through the uterus, but it may take longer.^9,10^ The medical abortion method has advantages over surgery: it could be offered on an outpatient basis, does not require hospitalization or anesthesia, and is more cost-effective than surgery.^11^ Misoprostol is a synthetic analog of prostaglandin E1 that was first developed to prevent gastric ulcers related to anti-steroid drugs. The efficacy and safety of misoprostol for incomplete abortion have been confirmed.^12^

The need for curettage usually arises if the patients have an incomplete abortion, heavy/prolonged vaginal bleeding, or continued pregnancy.^13^ Various methods are used to determine the effectiveness of medical abortion: gynecological self-assessment, clinical evaluation, ultrasound, and human chorionic gonadotropin (hCG) measurement in serum or urine.^14^ Transvaginal ultrasound imaging is a valuable tool for imaging uterus, ovaries, and pelvic floor space.^15^ Although endometrial thickness measurement by ultrasound has been used as a tool for predicting abortion failure, endometrial thickness prediction for uterine aspiration seems to be poorly used. Ultrasound findings may be difficult to interpret and may lead to unnecessary surgery. Serum gonadotropin level appears to be more effective than ultrasound scan at 14 days.^14^ Moreover, gonadotropin is a hormone produced by differentiated syncytiotrophoblast cells in early pregnancy and serves as the primary fetal signal to maintain pregnancy.^16^ Complete abortion is indicated by a decrease in β-hCG level during follow-up. Additionally, measuring β-hCG is easy and could be performed in most centers. Some women prefer transvaginal ultrasound imaging. β-hCG measurement is also useful in the diagnosis of pregnancy continuation, incomplete abortion, and ectopic pregnancy.^17^ Nearly one in four women experiences miscarriage during their lifetime. In many cases, the abortion is not complete and part of the fetus remains in the uterus.^18,19^ In Iran, 59% of abortions occur in 8–10 weeks of pregnancy.^20^ Attention to complete abortion has a major impact on maternal health in subsequent pregnancies.^21,22^

Comparison of the efficacy of ultrasound with β-hCG test showed that although serum β-hCG is as effective as ultrasound imaging in the diagnosis of completed abortion, β-hCG was more successful than ultrasound in the diagnosis of complete abortion at the second week in cases that had pain as a symptom.^2^ However, another study reported that measuring β-hCG is not as effective as ultrasound imaging in the diagnosis of completed abortion.^23^ Moreover, β-hCG level measurement is an efficient alternative to endometrial thickness transvaginal ultrasound measurement.^1^ The effectiveness of serum β-hCG and transvaginal ultrasound imaging in the diagnosis of incomplete abortion still needs investigation.^24^ Performing many diagnostic methods, including ultrasound and blood serum gonadotropin levels, can be expensive for both treatment systems and patients, and choosing an effective and cost-effective treatment method is critical. There are also few studies in the developing countries on this subject. Therefore, diagnostic evaluations to compare the mentioned methods could increase our knowledge in this interface. Therefore, the goal of this study is to compare the effectiveness of serum β-hCG and transvaginal ultrasound imaging in the determination of completed medical abortion.

## Materials And Methods

### Study design

This cross-sectional study was conducted in Ali Ibn Abi Talib, a referral educational hospital and a tertiary center of Sistan and Baluchestan Province in Zahedan, Iran. Furthermore, 285 women with a diagnosis of missed abortion confirmed using vaginal ultrasound with a crown-rump length of 10 mm and positive β-hCG tests, who were candidates for medical abortion between 2015 and 2017, participated in this study. This study was approved by the Ethics Committee of our university (No: IR.ZAUMS.REC.1393.6754).

### Inclusion and exclusion criteria

The inclusion criteria were (1) missed abortion or blighted ovum and (2) gestational age up to 12 weeks from the first day of the last menstrual period and confirmed by ultrasonography. The exclusion criteria were (1) fever greater than 38°C, (2) leukocytosis, (3) thrombocytopenia, (4) neutrophilia, (5) severe uncontrolled bleeding, (6) unstable hemodynamic, (7) anemia, (8) molar pregnancy, (9) infectious abortion, and (10) incomplete abortion.

### Study protocol

The study population was chosen at random and the study protocol was explained to all participants, and all of them signed an informed consent before joining the study. After taking participants’ medical history and performing physical examination, transvaginal ultrasound imaging (Medison 8000, 7.5 MHz vaginal probe) and β-hCG measurement were conducted to confirm abortion before beginning medical treatment. All women received 800 μg of vaginal misoprostol (Misoglandin 100 μg, Sami Saz Co., Iran). If the pregnancy products did not turn up with or without a little bleeding after 24 hours, a further 800 μg of vaginal misoprostol was administered.

The first assessment was carried out with transvaginal ultrasound imaging and serum β-hCG measurement after two weeks of medical attention. For transvaginal ultrasound, a qualified and skilled physician or advanced‐care clinician performed a sonographic examination, and images from all examinations were reviewed by a physician investigator (the physician was blind to the results), and for β-hCG measurement from patients, five to six cc of venous blood was taken after eight hours of fasting, and then β-hCG levels were checked. Abortion was deemed to be completed if β-hCG was negative or decreased by more than 80%. If the uterus was empty (did not display any residue) in the transvaginal ultrasound imaging or if the endometrial thickness was less than 15 mm, the abortion was considered completed.

In case the abortion was not confirmed to be completed in the second week, both tests were repeated in the fourth week. If the β-hCG measurement was negative in the fourth week and ultrasound imaging revealed no remains or endometrial thickness of less than 15 mm, the abortion was considered completed. Otherwise, curettage was planned. If the participant had severe bleeding 48 hours after admission, she was transferred to the operating room for curettage. In the absence of complications, all participants were discharged four hours after abortion and received information on abnormal symptoms such as fever, unpleasant weakness, vaginal bleeding, and pain before discharge and were also advised to return for a follow-up visit 14 days after discharge. All pregnant mothers were informed that this procedure had no effects and will not impose a cost on the patient.

Finally, a distinction was made between positive and negative findings of ultrasound imaging and β-hCG, and false- and true-negative and false- and true-positive findings were also calculated.

### Biomedical analysis

Venous blood samples were collected in test tubes containing clot activator, stored immediately on ice, and centrifuged at 3000 rpm for 12 minutes, 1 hour after the collection. Plasma was separated and stored at − 70°C for one hour to be analyzed.

Serum β-hCG was measured by an enzyme-linked immunosorbent assay with a commercial kit (Diaplus, Tina Pajoohan Arvin Co., Canada) for all participants. With this formula, β-hCG level was calculated based on the decrease percentage: β-hCG = (secondary β-hCG – primary β-hCG)/(primary β hCG) x 100 .The endometrial thickness was also calculated based on millimeters.

### Statistical analysis

All data were analyzed using the Statistical Package for the Social Sciences. Descriptive analysis was used to calculate the mean and variance. The Kappa coefficient was determined to measure the agreement between β-hCG measurement and transvaginal ultrasound imaging. In addition, the p value of less than 0.05 was considered significant. The agreement between the two tests and their sensitivity and specificity were compared.

## Results

Further, 288 women underwent medical abortion in our hospital. They were all chosen to take part in our study. However, six of them were excluded at the start of the study because it was found out that they had the exclusion criteria. Also, seven other participants were excluded as they did not complete the follow-up protocol of the study. As a result, 275 women completed this study. The mean age of women was 27.9 ± 6.0 years old, while the mean weight was 70.03 ± 17.25 kg. Additionally, the mean gestational age was 12 ± 1.4 weeks. The number of prior pregnancies was evaluated, and it was found that the mothers had 2.1 ± 0.8 births.

**Figure 1.**
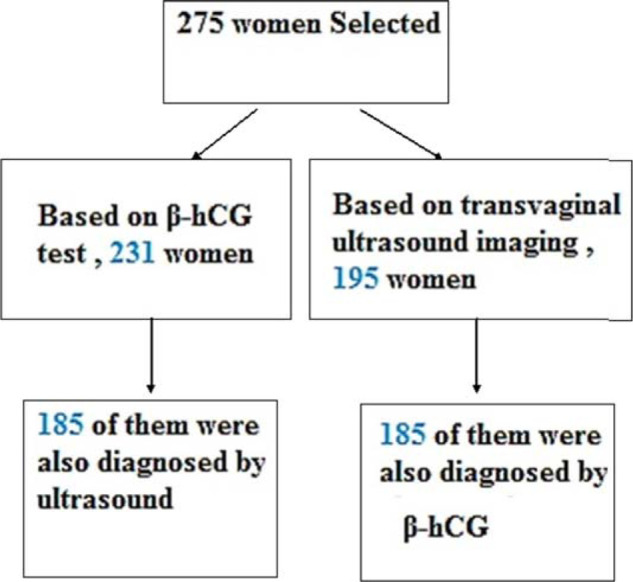
Study flow chart of the 275 women who underwent a medical abortion, 231 were diagnosed using β-hCG, and 195 of them were identified by transvaginal ultrasound

Based on β-hCG test results, 231 women (84.3%) had completed the medical abortion in the second week following treatment. By the fourth week, all of them had completed the medical abortion. Based on transvaginal ultrasound imaging results, 195 women (70.8%) had completed the medical abortion in the second week following treatment. By the fourth week, all had completed the medical abortion (Figure 1).

Among 195 women whose abortion had been confirmed by transvaginal ultrasound imaging in the second week, 185 of them (95.2%) were also diagnosed by β-hCG in the second week (Figure 1). In the same way, 185 of the 231 women (80%) whose abortion had been approved by β-hCG in the second week were also diagnosed using ultrasound imaging in the second week. The Kappa agreement coefficient for the two methods was 0.434 in the second week (Table 3; *p* < 0.0001).

Ultrasound findings revealed 108 true positives and 54 false negatives; 54 false negatives diagnosed by ultrasound were attributed to women who had an abortion and where ultrasound could not detect it. Thus, the sensitivity of ultrasound in diagnosis of abortion was calculated to be 68.5. In the feature, there were 20 true negatives and 11 false positives. In addition, there were 11 false positives diagnosed by ultrasound in non-abortion women who mistakenly had a complete miscarriage, so the vaginal ultrasonography specificity was estimated to be 64.5%. Among the patients, 251 had a complete medical abortion, but 195 of them were detected by ultrasound. Thus, the positive predictive value for abortion diagnosis was calculated to be 77%. Among the patients studied, 24 mothers did not have a complete abortion, but the ultrasound diagnosed 80 patients as healthy. So the negative predictive value was calculated to be 30%.

Findings of β-hCG measurement evaluation showed 215 true positives and 9 false negatives. The nine false-negative cases diagnosed by β-hCG were linked to women who had had an abortion and were not diagnosed. Thus, the sensitivity was calculated to be 95.9%. There were 31 true negatives and 5 false positives in the feature. Thus, the specificity of β-hCG measurement was calculated to be 86%.

Among the patients, 251 had a complete medical abortion, but the β-hCG measurement identified 231 as complete abortions, which resulted in positive predictive value of 80%. Moreover, 24 patients had no abortion, but β-hCG measurement had diagnosed 34 patients. As a result, a negative predictive value of 70% was calculated. However, β-hCG measurement evaluation findings provided higher levels of confidence in medical abortion detection (Table 3).

Findings of T-test statistical analysis have shown that the two methods studied had statistically significant differences in terms of sensitivity, specificity, and negative news value (Table 4).

## Discussion

In our study focused on β-hCG, 84.3% of cases had completed abortion in the second week after treatment, and all of individuals had completed abortion in the fourth week. In a similar study, Pocius and colleagues evaluated the β-hCG decrease in the first trimester of abortion. The β-hCG decreased by 56.9% in the third day, 73.5% in the fourth day, and 92.9% in the sixth day. The percentage of completed abortion without further intervention was 93%.^25^

Ultrasound and human chorionic gonadotropin (hCG) measurement in serum or urine are some of the methods mainly used to evaluate the effectiveness of medical abortion.^14^ There are various reports on the use of these two methods in the diagnoses of completed abortion. In the present study, completed abortion was confirmed in 231 women with β-hCG measurement and in 195 women with ultrasound imaging in the second week. Thus, the success percentage of β-hCG measurement was 95.2% compared to ultrasound imaging in detecting completed abortion in the second week.

During the study, there were deviations from the protocol, the reasons for which were abnormalities in the studies performed, unsuccessful ultrasound, and inconsistency with the patient. Another limitation of the study is the lost percentage for follow-up.

There is no theoretical consensus on the threshold for hCG. There are also different opinions on the rate of decrease in blood hormones following medical abortion. Some authors believe that this rate depends on the initial level of hCG and shows a faster decrease when the baseline concentration is higher at the start of treatment. In addition, the reported reduction was as follows: from 21% to 35% in two days, from 60% to 84% in seven days, and depending on the initial amount of hCG with 90% reduction in 95% of women in five days^14^

Based on the results of a study by Behnamfar et al., β-hCG is as successful as ultrasound in confirming medical abortion in early pregnancy but should be used as a complementary method in clinical evaluations.^2^ On the other hand, Nasaf et al. stated that β-hCG level measurement is an effective alternative to endometrial thickness transvaginal ultrasound measurement to confirm completion of early termination of abortion.^1^ Reeves et al. demonstrated that endometrial thickness is not a clinically useful tool to predict this unwanted outcome. Regardless of the endometrial thickness threshold used, the PPV did not exceed 25%. Additionally, the sensitivity and specificity values obtained confirm that endometrial thickness is a poor test to predict the subsequent need for medical abortion and curettage.^26^

However, another study has shown that evaluating completed abortion by measuring β-hCG does not decrease unplanned interventions and visits relative to ultrasound imaging. However, given that the number of unplanned interventions in both methods is low, the patients would accept both.^23^ These findings are not consistent with our results, which might be because of the study design.

Peng and colleagues also reported that the major limitation of β-HCG testing is the frequent changes in hormone during early pregnancy, whereas transvaginal ultrasound and serum β-HCG assays could overcome serum β-HCG assay concentrations.^27^

β-HCG is produced by syncytiotrophoblast cells and reflects the activity of villus. Continuous detection of blood β-HCG could identify intrauterine pregnancy and ectopic pregnancy.^10,28,29^ The serum β-HCG level could directly reflect the viability of trophoblasts, and the high β-HCG level indicates the high proliferation activity of trophoblasts and high invasion into fallopian tube. Moreover, dynamic monitoring of serum β-HCG changes can predict the prognosis of tubal pregnancy. If the serum β-HCG level is < 2,000 IU/l, the drug therapy and non-surgical conservative treatment could be chosen, and if the serum β-HCG level continues to rise >8,000 IU/l, the rupture of tubal pregnancy should be identified and treated by surgery as early as possible.^30–32^

We found out that β-hCG measurement is more effective in diagnosing completed abortion and the sensitivity, specificity, and positive and negative predictive values of β-hCG in the second week were 95.2%, 86.7%, 84%, and 70, respectively, which were significantly higher than the ultrasound method. A similar study reported the sensitivity of 90%, specificity of 70.8%, positive predictive value of 56.4%, and negative predictive value of 94.4%, with the diagnostic accuracy of 83.5%.^1^

In different studies, these two methods have been compared, and complementary role of these two methods has been mentioned together. According to a similar study, although β-hCG measurement is a good alternative to ultrasound in pursuing abortion medical treatment, the two methods are not superior to each other and are equivalent.^24^ The results of another study also showed that ultrasound and β-hCG level measurement are useful in predicting abortion but should always be accompanied by clinical evaluation.^33^ Lewis et al. stated in their studies that serum hCG measurement < 900 IU/l, 14–21 days following medical abortion, is an efficient strategy for excluding ongoing pregnancy after a first trimester of medical abortion.^14^

Based on our results and the other mentioned studies, β-hCG measurement could be a reliable method to diagnose completed medical abortion in two weeks following treatment. Finally, it is recommended to use this method as one of the diagnostic strategies for recognizing the full potential of abortion in health centers, given the sensitivity of this method in identifying cases of abortion and its cost.

A limitation of our study was that some participants did not cooperate easily. The negative attitude of some mothers toward such research projects was one of the reasons for this limitation and lack of cooperation, though explaining the nature of the research led to their greater cooperation in all stages of the study. Although we assessed the aspects of β-hCG and transvaginal ultrasound, we did not evaluate the predictive value of other methods, and examining these methods in future clinical trials could be useful.

## Conclusion

The diagnosis and confirmation of completed medical abortion was properly performed by β-hCG measurement and ultrasound. Our study showed that β-hCG measurement is just as effective as ultrasound to confirm successful medical abortion in early pregnancy but should be used as a supplement to clinical evaluations. Therefore, it is suggested that β-hCG measurement and clinical examination be the first choice of diagnosis, and ultrasound imaging could be used whenever it was necessary. Also, it is recommended that the effect of β-hCG measurement and ultrasound imaging be compared in diagnosing complete abortions in multicentered research with a greater number of participants.

### Disclosure

This study is based on a residency dissertation funded by our university.

### Acknowledgments

We are indebted to all patients who participated in this research.

## Figures and Tables

**Table 1:** Demographic information of the subjects

Parameters	(n = 275)

Age (year)	27.9 ± 6.0

Weight(kg)	70.03 ± 17.25

Gestational age (week)	12 ± 1.4

Number of previous pregnancies	2.1 ± 0.8


**Table 2:** Comparing β-hCG measurement and ultrasound imaging in confirming completed abortion

Confirming abortion method		Ultrasound imaging		Total	Kappa coefficient

		Yes	No		

β-hCG measurement	Yes	185 (95.2%)	46 (57.7%)	231 (84.3%)	0.434

	No	10 (4.8%)	34 (42.3%)	44 (15.7%)	

Total		195 (100%)	80 (100%)	275 (100%)	

		Total disease	Total non-disease		

Total confirming		251 (91.2%)	24 (8.8%)	275 (100%)	


**Table 3:** Sensitivity, specificity, and negative predictive value

Character	Assessment of β-hCG	Transvaginal ultrasound

Sensitivity	95.2%	68.5%

Specificity	86.7%	64.5%

Positive predictive	84%	77%

Negative predictive	70%	30.%


**Table 4:** Statistical comparison of the two methods studied

			95% CI	

	Cohort	*p* value	Lower bound	Upper bound

β-hCG sensitivity	Ultrasound sensitivity	0.034	− 4.91	32.40

β-hCG specificity	Ultrasound specificity	0.013	− 0.24	28.72

β-hCG positive predictive	Ultrasound positive	0.105	4.71	19.14

β-hCG negative predictive	Ultrasound negative	0.040	6.71	69.14

Ultrasound sensitivity	β-hCG sensitivity	0.034	− 32.40	4.91

Ultrasound specificity	β-hCG specificity	0.013	− 28.72	0.24

Ultrasound positive	β-hCG positive predictive	0.105	− 19.14	− 4.71

Ultrasound negative	β-hCG negative predictive	0.040	− 69.14	− 6.71

